# Stromal and epithelial syndecan-1 expression in benign and malignant salivary gland tumors: which is more reflective of behavior?

**DOI:** 10.1016/j.bjorl.2019.07.006

**Published:** 2019-09-04

**Authors:** Mojgan Alaeddini, Farzad Yazdani, Shahroo Etemad-Moghadam

**Affiliations:** a Tehran University of Medical Sciences, Dentistry Research Institute, Dental Research Center, Tehran, Iran; b Tehran University of Medical Sciences, Amiralam Hospital, Department of Pathology, Tehran, Iran

**Keywords:** Syndecan-1, Salivary gland neoplasms, Stroma

## Abstract

**Introduction:**

Salivary gland tumors are a diverse group of lesions, with various origins and extremely different behaviors, leading to a variety of outcomes for patients. Therefore, the need to discover novel markers with the ability to predict the behavior of benign and malignant salivary gland neoplasms is crucial. Syndecan-1 is a cell-surface protein with significant roles in various aspects of tumor function. Its expression in salivary gland neoplasms, especially their stromal component, has not been investigated.

**Objectives:**

We aimed to assess the immunopositivity of syndecan-1 in epithelial and stromal components of salivary gland neoplasms and to compare it between benign and malignant subtypes in addition to evaluating its correlation with clinicopathologic parameters.

**Methods:**

133 salivary gland tumors were immunohistochemically stained with syndecan-1 and the intensity and percentage of this protein was determined, compared between the tumors and correlated with clinicopathologic factors.

**Results:**

Statistical analysis of lesions with a sufficient sample size showed significant differences in percentage and intensity between both epithelial and stromal components of all tumors (*p* < 0.05). Pairwise-comparisons demonstrated significantly higher staining-percentage of epithelial cells (*p* = 0.02) in Warthin’s tumor compared to pleomorphic adenoma and adenoid cystic carcinoma. Similarly, significantly higher staining intensities and/or percentages was observed in mucoepidermoid carcinoma and adenoid cystic carcinoma compared to pleomorphic adenoma and Warthin’s tumor (*p* < 0.05). Of the clinicopathologic factors, there was only a significant negative correlation between stromal percentage of mucoepidermoid carcinoma and age and a significant difference between stromal intensity+percentage of adenoid cystic carcinoma and gender (*p* < 0.05).

**Conclusions:**

According to our findings we postulate that stromal syndecan-1 correlates with the behavior of salivary gland tumors, with malignant neoplasms demonstrating a higher expression, indicating a role for syndecan-1 in invasion and metastasis.

## Introduction

The salivary system is composed of three bilateral pairs of major salivary glands (parotid, submandibular and sublingual) and hundreds of minor salivary glands located and spread in the upper respiratory tract.^1^ Tumors arising from these tissues exhibit a considerable histological variation and are classiﬁed into benign and malignant neoplasms with variable aggressiveness and a potential to develop metastasis.^2^ Since normal salivary glands have significantly different types of cells, salivary gland tumors can originate from any one of these cell types.^1^ This cellular diversity and overlapping histologic features cause difficulties in the diagnosis and understanding of the pathogenesis of these tumors. In recent years, molecular evaluation and identification of cell-biological details have been used for more precise diagnoses and treatment of tumors.^3^ Previous research has identified various molecular markers responsible for the development and progression of salivary gland tumors.^1^, ^3^, ^4^ A better perception of the cellular and molecular alterations in salivary gland tumors can provide an insight into molecular pathogenesis, diagnosis, and treatment of these neoplasms. Several studies of salivary gland tumors have investigated the involvement of different markers in various aspects of tumorigenesis, such as proliferation, apoptosis, cell migration, cell cycle regulation and metastasis.^1^, ^5^, ^6^

The exact and accurate adhesion of cells to each other and their surroundings depends on the presence and function of different cell adhesion molecules.^7^ Syndecan-1 (CD138) is a member of the cell surface heparan sulphate proteoglycan family which is involved in multiple cellular events by binding to various growth factors and extracellular matrix components.^8^ This protein has an essential role in epithelial homeostasis, cell morphology, differentiation, proliferation, and migration. In recent years, a number of studies have suggested that syndecan-1 not only contributes to normal cellular biological phenomena but also plays a role in different pathological processes, including benign tumors and cancers.^9^ Despite the fact that syndecan-1 reduction has been reported during malignant transformation in several cancers, some malignancies such as non-Hodgkin lymphoma, malignant glioma, and pancreatic cancer show increased CD138 expression.^10^, ^11^, ^12^, ^13^ This evidence exhibits contradictory functions of syndecan-1 in the pathobiology of different tumors. To our knowledge, there have been limited studies on the expression of syndecan-1 in salivary gland tumors and little information is available about its expression pattern in these lesions.^14^, ^15^ The purpose of this research was to evaluate and compare the immunoexpression of syndecan-1 in a variety of benign and malignant salivary gland tumors and to correlate its expression with clinicopathologic parameters.

## Methods

133 formalin-fixed paraffin-embedded blocks of salivary gland tumors were obtained from the archives of Amir Alam Pathology Department. These neoplasms included 30 pleomorphic adenomas, 30 Warthin’s tumors, 2 basal cell adenomas, 2 myoepithelioma, 30 adenoid cystic carcinomas, 30 mucoepidermoid carcinomas and 9 acinic cell carcinomas.

Two pathologists reviewed the slides and the diagnoses of all tumors were confirmed. Mucoepidermoid carcinoma samples were graded according to the Brandwein grading system^16^ and adenoid cystic carcinoma was categorized as cribriform, tubular and solid based on morphologic features. The demographic and clinical data of all patients including age, gender, location, tumor size, lymph node metastasis and neuroinvasion were also retrieved.

The protocol of this research was approved by the Ethics Committee of our University (approval nº 1396.2164; date: 4/30/2017). For immunohistochemistry, 3-micrometer paraffin-embedded tissue sections were spread on 3 poly-L-lysine coated slides, and were subsequently immersed in various degrees of alcohol and rehydrated. Antigen retrieval was done by incubating (14 min) the samples with citrate buffer (PH, 6.0) in a microwave. The sections were treated with 0.3% hydrogen peroxide (10 min) for abolishing endogenous peroxidases activity and were then exposed to the primary monoclonal antibody anti-human syndecan-1 (clone MI15 Dako Corporation, Carpinterıa, CA, USA, dilution 1:40) at room temperature (1 h). Finally, the EnVision System (Dako Cytomation, Glostrup, Denmark) was used in this study for revealing antibody staining at room temperature (30 min). Positive controls consisted of human tonsil tissue and the slides for negative control were incubated without the primary antibody.

The percentage of positive cancer cells and tumor stroma were separately assessed as follows: (0) negative, (1) 1%–10%, (2) 11%–50% and (3) > 50%. Intensity of the stained cells was scored as low, intermediate and high.^17^

Re-confirmation of the initial diagnosis and all immunihistochemical scorings were performed by two pathologists, using a double-headed microscope and any disagreements were resolved by consensus.

In this study, Spearman, Kruskal-Wallis and Mann Whitney U tests adjusted for multiple comparisons were used. *p*-values < 0.05 were considered statistically significant.

## Results

Normal salivary glands adjacent to tumors exhibited low to intermediate staining of syndecan-1 in some ductal tissues. The tumors were evaluated from two aspects, neoplastic and stroma cells.

**Figure 1:**
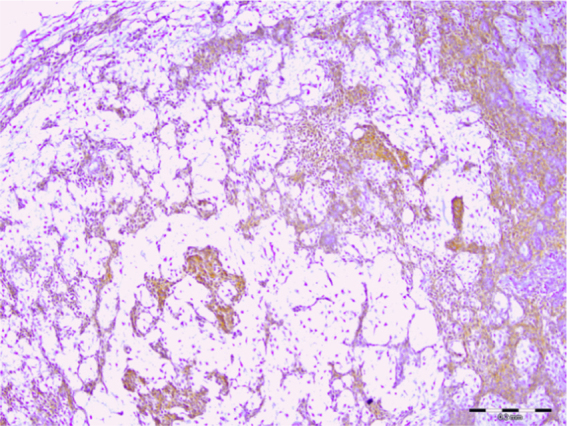
Immunohistochemical expression of syndecan-1 in pleomorphic adenoma (scale bar represents 0.2 mm).

**Figure 2:**
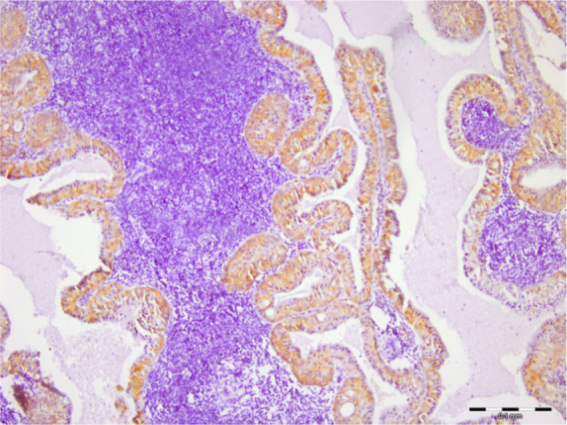
Syndecan-1 staining in Warthin’s tumor (scale bar represents 0.1 mm).

All (100%) of the 30 pleomorphic adenomas showed expression of syndecan-1 in tumor cells and the staining was localized to the cytoplasmic and membranous components of ductal and non-ductal neoplastic cells. However, 20 (67%) did not show any expression of this protein in the stroma and the rest showed a low level of immunopositivity (Fig. 1). As for Warthin’s tumors, the expression of syndecan-1 in neoplastic cells and lymphoid stroma was seen in 100% and 26% of the samples, respectively. Cytoplasmic and membranous localization of syndecan-1 expression was found in the bi-layered oncocytic epithelium (Fig. 2). We observed high syndecan-1 expression in tumor cells in two cases of basal cell adenoma and myoepithelioma. However, stromal expression of CD138 in these tumors was low.

**Figure 3:**
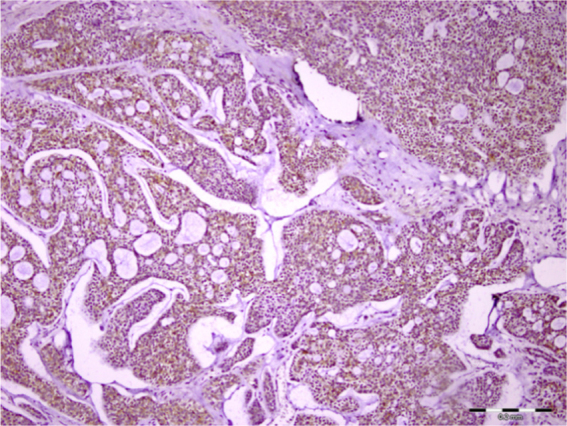
Syndecan-1 immunoreactivity was found in stroma and tumor cells of adenoid cystic carcinoma (scale bar represents 0.2 mm).

**Figure 4:**
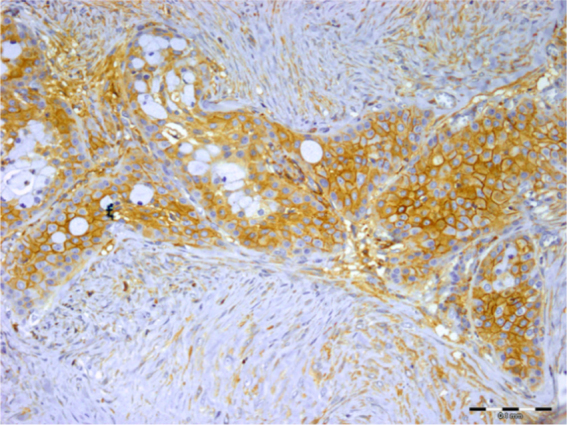
Immunostaining of syndecan-1 was observed in tumor and stromal cells, but not in mucous cells of mucoepidermoid carcinoma (scale bar represents 0.1 mm).

Regarding adenoid cystic carcinoma, 97% and 54% of the samples showed immunostaining in tumor cells and in the stroma, respectively. This expression was most prominently seen in the cell cytoplasm (Fig. 3). Syndecan-1 was expressed in both epithelial and stromal components of all mucoepidermoid carcinomas (100%). Mucous cells were not stained in most of the samples, and immunopositivity in tumor cells was mostly cytoplasmic and sometimes membranous (Fig. 4). All nine (100%) acinic cell carcinoma cases were positive for syndecan-1 both in the stroma and cancer cells. Cytoplasmic staining was observed in most cells with variable intensity. Similar to acinic cell carcinoma, all three parotid SCC specimens (100%) showed staining for CD138. Percentage and intensity of syndecan-1 in epithelial and stromal components of all studied neoplasms are demonstrated in Table 1.

**Table 1:** Percentage and intensity of syndecan-1 in epithelial and stromal components of benign and malignant salivary neoplasms.

		Percentage				Intensity				
	N	1	2	3	4	0	Low	Intermediate	High	
Pleomorphic adenoma	Epithelial	30	0	3	10	17	0	9	18	3
	Stromal	30	20	10	0	0	20	10	0	0
Warthin’s tumor	Epithelial	30	0	0	3	27	0	9	14	7
	Stromal	30	22	7	1	0	22	3	4	1
Mucoepidermoid carcinoma	Epithelial	30	0	3	4	23	0	0	14	16
	Stromal	30	0	1	19	10	0	0	19	11
Adenoid cystic carcinoma	Epithelial	30	1	4	7	18	1	9	17	3
	Stromal	30	11	14	4	1	11	12	6	1
Acinic cell carcinoma	Epithelial	9	0	5	2	2	0	6	3	0
	Stromal	9	0	5	4	0	0	5	4	0
Basal cell adenoma	Epithelial	2	0	0	1	1	0	1	1	0
	Stromal	2	0	2	0	0	0	2	0	0
Myoepithelioma	Epithelial	2	0	0	2	0	0	0	2	0
	Stromal	2	0	2	0	0	0	2	0	0

Statistical analyses were carried out only in lesions with a sufficient sample size, including pleomorphic adenoma, Warthin’s tumor, adenoid cystic carcinoma and mucoepidermoid carcinoma; a description was provided for the remaining tumors.

The Kruskal-Wallis test showed a significant difference in the percentage and intensity of syndecan-1 expression in tumor cells among the evaluated lesions (*p* = 0.009 and *p* < 0.001 respectively). Paired comparison with Mann–Whitney U test revealed that only CD138 percentage of Warthin’s tumor cells was significantly higher compared to pleomorphic adenoma and adenoid cystic carcinoma (both, *p* = 0.02).

A significant difference was also found in staining intensity and percentage of syndecan-1 in the stroma of these tumors (both, *p* < 0.001). According to pairwise comparisons (Mann-Whitney), a significantly lower expression of syndecan-1 percentage was observed in the stroma of pleomorphic adenoma compared to adenoid cystic carcinoma (*p* = 0.04) and syndecan-1 intensity+percentage in mucoepidermoid carcinoma (both, *p* < 0.001). Moreover, in Warthin’s tumor, stromal syndecan-1 percentage was lower than that in adenoid cystic carcinoma (*p* = 0.02) and both intensity and percentage were reduced as compared to mucoepidermoid carcinoma (both, *p* < 0.001). There was a significant difference in the stromal percentage and intensity of syndecan-1 between mucoepidermoid carcinoma and adenoid cystic carcinoma (both, *p* < 0.001), with higher immunoreactivity observed in the former.

There was no correlation between expression of syndecan-1 in malignant cells and the stroma of these tumors (*p* = 0.79). We also analyzed the association of CD138 expression with clinicopathological factors in both tumor cells and stroma of the studied neoplasms. The Spearman test showed a statistically significant negative correlation between stromal percentage of syndecan-1 and age in the mucoepidermoid carcinoma group (*p* = 0.01, r = −0.43). We also found a significant difference in the staining intensity and percentage of this protein in the stroma between male and female patients in adenoid cystic carcinoma samples (*p* = 0.02 and *p* = 0.03, respectively). There was no significant correlation between the other clinicopathological parameters and syndecan-1 expression in the studied tumors (Table 2, Table 3).

**Table 2:** Syndecan-1 expression in malignant salivary gland tumors according to available clinicopathologic variables.

Variables	Mucoepidermoid carcinoma																Adenoid cystic carcinoma															
	Epithelial component								Stromal component								Epithelial component								Stromal component							
	Intensity				Percentage				Intensity				Percentage				Intensity				Percentage				Intensity				Percentage			
	0	1	2	3	1	2	3	4	0	1	2	3	1	2	3	4	0	1	2	3	1	2	3	4	0	1	2	3	1	2	3	4
Age																																
<50	0	0	6	9	0	1	2	12	0	0	8	7	0	0	7	8	1	2	8	1	1	1	3	7	5	4	3	0	5	4	3	0
≥50	0	0	7	7	0	1	2	11	0	0	10	4	0	1	11	2	0	7	8	2	0	3	4	10	6	8	2	1	6	9	1	1
Location																																
Minor	0	0	0	1	0	0	0	1	0	0	0	1	0	0	1	0	1	6	13	1	1	2	6	12	7	11	3	0	7	12	2	0
Major	0	0	13	15	0	2	4	22	0	0	18	10	0	0	1	17	0	3	4	2	0	2	1	6	4	1	3	1	4	2	2	1
Gender																																
F	0	0	7	9	0	1	2	13	0	0	10	6	0	0	12	4	0	6	8	1	0	1	7	7	8	5	2	0	8	6	1	0
M	0	0	6	7	0	1	2	10	0	0	8	5	0	1	6	6	1	3	9	2	1	3	0	11	3	7	4	1	3	8	3	1
Size																																
≤2 cm	0	0	2	4	0	0	0	6	0	0	5	1	0	0	4	2	0	0	2	0	0	0	1	1	1	1	0	0	1	1	0	0
>2 cm	0	0	10	8	0	2	3	13	0	0	11	7	0	1	11	6	1	9	14	3	1	4	6	16	10	11	5	1	10	12	4	1
LNM/NI^a^																																
1	0	0	2	3	0	1	1	3	0	0	4	1	0	0	5	0	1	6	8	3	1	3	3	11	6	8	3	1	6	9	2	1
2	0	0	10	9	0	1	2	16	0	0	12	7	0	1	10	8	0	2	8	0	0	1	3	6	4	4	2	0	4	4	2	0
Pattern/ Grade^b^																																
1	0	0	8	10	0	1	3	14	0	0	11	7	0	0	11	7	1	7	14	2	1	3	6	14	9	10	5	0	9	11	4	0
2	0	0	3	3	0	0	1	5	0	0	3	3	0	0	4	2	0	2	0	1	0	1	1	1	1	1	0	1	1	1	0	1
3	0	0	2	3	0	1	0	4	0	0	4	1	0	1	3	1	0	0	3	0	0	0	0	3	1	1	1	0	1	2	0	0

a 1 and 2 represent positive and negative Lymph Node Metastasis (LNM) for mucoepidermoid carcinoma and positive and negative Neuroinvasion (NI) for adenoid cystic carcinoma, respectively.

b 1, 2 and 3 represent low, intermediate and high grades for mucoepidermoid carcinoma and cribriform, solid and tubular histologic patterns for adenoid cystic carcinoma, respectively.

**Table 3:** Syndecan-1 immunopositivity in benign salivary gland tumors according to available clinicopathologic variables.

Variables	Pleomorphic adenoma																Warthin’s tumor															
	Epithelial component								Stromal component								Epithelial component								Stromal component							
	Intensity				Percentage				Intensity				Percentage				Intensity				Percentage				Intensity				Percentage			
	0	1	2	3	1	2	3	4	0	1	2	3	1	2	3	4	0	1	2	3	1	2	3	4	0	1	2	3	1	2	3	4
Age																																
<50	0	8	10	2	0	3	6	11	13	7	0	0	13	7	0	0	0	1	3	1	0	0	1	4	2	1	1	1	2	3	0	0
≥50	0	1	8	0	0	0	4	5	7	2	0	0	7	2	0	0	0	8	11	6	0	0	2	23	20	2	3	0	20	4	1	0
Location																																
Minor	0	0	0	1	0	0	0	1	1	0	0	0	1	0	0	0	0	0	0	0	0	0	0	0	0	0	0	0	0	0	0	0
Major	0	9	18	1	0	3	10	15	19	9	0	0	19	9	0	0	0	9	14	7	0	0	3	27	22	3	4	1	22	7	1	0
Gender																																
F	0	4	10	1	0	2	5	8	9	6	0	0	9	6	0	0	0	0	0	0	0	0	0	0	0	0	0	0	0	0	0	0
M	0	5	8	1	0	1	5	8	11	3	0	0	11	3	0	0	0	9	14	7	0	0	3	27	22	3	4	1	22	7	1	0
Size																																
≤2 cm	0	2	2	0	0	2	0	2	3	1	0	0	3	1	0	0	0	2	0	0	0	0	1	1	1	0	1	0	1	1	0	0
>2 cm	0	7	15	2	0	1	9	14	16	8	0	0	16	8	0	0	0	7	14	7	0	0	2	26	21	3	3	1	21	6	1	0

## Discussion

Despite many reports on the roles of syndecan-1 in the tumorigenesis of different kinds of neoplasms,^9^, ^10^, ^11^ the expression and functional details of this protein in salivary gland tumors is not clear. Most of the investigations on syndecan-1 in non-salivary gland tumors have focused on its expression in neoplastic cells^18^, ^19^, but, recent studies suggest that CD138 may have a role in the tumor stroma of some neoplasms.^20^, ^21^ To our knowledge, there is no former research on the expression of CD138 in the stroma of salivary gland tumors. Interestingly, some studies on the stroma of various cancers have shown a different role for syndecan-1 in the stroma compared to the epithelium. In contrast to decreased expression of this protein in malignant cells, its increased expression in the stroma is associated with invasion and metastasis.^20^, ^21^, ^22^, ^23^ In the present study, for the first time, we evaluated the expression of CD138 in the stroma of salivary gland tumors.

Previous studies have shown that high stromal expression of syndecan-1 is not a common finding in normal and non-neoplastic processes, except in cases like wound healing and tooth development.^23^, ^24^ When mesenchymal-epithelial interactions in dental development occur, the expression of this protein increases in the mesenchyme and decreases in the epithelium during a specific period of time. This transient event appears to be related to the induction effect of the epithelium.^24^ The source of syndecan-1 expression in tumor stroma is not clear and accordingly various probabilities have been raised. For example the ectodomain can be released from the cell membrane or it may directly originate from the stroma itself.^25^ We showed a significant difference in the expression of syndecan-1 between both neoplastic cells and stroma of the studied salivary gland tumors. Based on the results of the current investigation, the expression of CD138 in the stroma of malignant tumors, *i.e.* mucoepidermoid carcinoma and adenoid cystic carcinoma was significantly higher compared to benign neoplasms, including pleomorphic adenoma and Warthin’s tumor. The findings of a study by Lendorf et al.^26^ on benign and carcinomatous breast lesions were in line with our study, indicating that the expression of this protein in the stroma of malignant neoplasms was higher than in benign tumors.

Evaluation of the stromal expression of syndecan-1 in some tumors has shown that CD138 plays an important role in invasion and progression of cancers, and it is essential to regulate the interaction between the epithelium and stroma of the tumor.^20^, ^21^, ^22^, ^23^ Syndecan-1 has the ability to bind to various proteins including Hepatocyte Growth Factor (HGF), vascular endothelial growth factor and fibroblast growth factor-2, and thus can potentially affect the growth of cancer cells and tumor progression by stimulating angiogenesis.^23^ Various studies have suggested that the CD105 mean vascular density, a marker of angiogenesis, is significantly higher in malignant salivary gland tumors versus benign neoplasms.^27^, ^28^ Interestingly, evidence suggests that high expression of syndecan-1 is associated with an increase in microvasculature density.^29^ This could help explain the increased expression of stromal CD138 in malignant tumors found in the present study. Considering the role of syndecan-1 in angiogenesis; this protein may contribute to increased angiogenesis in malignant salivary lesions.

On the other hand, Cardoso et al.^27^ found that mean vascular density was significantly higher in mucoepidermoid carcinoma compared to adenoid cystic carcinoma. Interestingly, in the present study, the expression of CD138 in the stroma of mucoepidermoid carcinoma was higher than its expression in adenoid cystic carcinoma, which may be due to the role of this protein in the regulation of angiogenesis between the two cancers.

Drexon et al.^30^ studied multiple myeloma cells and reported that CD138 plays an important role in the promotion of Met signaling through binding to HGF. They concluded this would increase cancer cell proliferation. Tsukinoki et al.^31^ emphasized that binding of stromal HGF to c-Met may lead to the aggressive growth and metastasis of high grade salivary carcinomas. Considering our finding of the increased expression of syndecan-1 in the stroma of malignant salivary gland tumors, it may be possible that one of the mechanisms through which this protein can impact the biology and prognosis of salivary gland tumors is its influence on the signaling of HGF and c-MET.

All tumors examined in this study showed the expression of syndecan-1 in tumor cells, suggesting that CD138 may contribute to the pathogenesis of salivary gland tumors. There was no significant difference in the expression of this protein in tumor cells between pleomorphic adenoma and malignant tumors, while a significant difference was observed between Warthin’s tumor and pleomorphic adenoma and adenoid cystic carcinoma. Therefore, the expression of this protein in tumor cells may not clearly reflect the difference in biological behavior between benign and malignant salivary gland tumors.

The only correlation between syndecan-1 and clinicopathologic factors was found in adenoid cystic carcinoma, where both intensity and percentage were higher in the stroma of male patients, which was similar to the findings of another study in colorectal carcinoma.^32^ We also found the percentage of this protein to be significantly higher in the stroma of younger patients (<50) with mucoepidermoid carcinoma. There is no accurate interpretation or clear explanation for these associations, indicating the need for more extensive and detailed research in this field.

In future investigations, tumors that had a small sample size in this study should be replaced with sufficient numbers of the same lesions, for more precise comparisons. On the other hand, to better understand the effect of this protein on the pathogenesis and invasion of salivary gland tumors, it is suggested that angiogenesis and other markers such as HGF and c-MET be investigated simultaneously with CD138.

## Conclusions

Syndecan-1 seems to play a role in the pathogenesis of salivary gland tumors. However, its immunoreactivity in the stroma of these neoplasms compared to tumor cells may be more involved in the biological behavior and invasion of these tumors.

## Funding

This work was supported by Dental Research Center, Dentistry Research Institute, Tehran University of Medical Sciences (TUMS) [grant number 96.469.14].

## Ethical approval

This article does not contain any studies with human participants or animals performed by any of the authors. The project of this study was approved by TUMS Ethics Committee (approval number IR.TUMS.DENTISTRY.REC.1396.2164; date: 4/30/2017).

## Conflicts of interest

The authors declare no conflicts of interest.
